# Two or Three? Clinical and Proteomic Perspectives on Dolutegravir/Lamivudine Versus Bictegravir/Emtricitabine/Tenofovir Alafenamide as Initial HIV Treatment

**DOI:** 10.1093/ofid/ofaf626

**Published:** 2025-10-06

**Authors:** Claudio Díaz-García, Sergio Serrano-Villar, Alejandro G. García-Ruiz de Morales, Robert Güerri-Fernández, Juncal Pérez-Somarriba, Sonsoles Sánchez Palomino, Inés Suárez-García, Cristina Hernández Gutiérrez, David Dalmau Juanola, Santiago Moreno, Elena Moreno, Javier Martínez-Sanz

**Affiliations:** Department of Infectious Diseases, Hospital Universitario Ramón y Cajal, IRYCIS, Madrid, Spain; CIBER de Enfermedades Infecciosas (CIBERINFEC), Instituto de Salud Carlos III, Madrid, Spain; Department of Medicine, Universidad de Alcalá, Madrid, Spain; Department of Infectious Diseases, Hospital Universitario Ramón y Cajal, IRYCIS, Madrid, Spain; CIBER de Enfermedades Infecciosas (CIBERINFEC), Instituto de Salud Carlos III, Madrid, Spain; Facultad de Medicina, Universidad Antonio de Nebrija, Madrid, Spain; Department of Infectious Diseases, Hospital Universitario Ramón y Cajal, IRYCIS, Madrid, Spain; CIBER de Enfermedades Infecciosas (CIBERINFEC), Instituto de Salud Carlos III, Madrid, Spain; CIBER de Enfermedades Infecciosas (CIBERINFEC), Instituto de Salud Carlos III, Madrid, Spain; Sección de Enfermedades Infecciosas, Hospital del Mar Research Institute, Group for Research in Viral Infections (GRIV), Barcelona, Spain; Medicine and Life Sciences Department (MELIS), Universitat Pompeu Fabra, Barcelona, Spain; CIBER de Enfermedades Infecciosas (CIBERINFEC), Instituto de Salud Carlos III, Madrid, Spain; Hospital Clínico San Carlos, Unidad de Enfermedades Infecciosas, Madrid, Spain; CIBER de Enfermedades Infecciosas (CIBERINFEC), Instituto de Salud Carlos III, Madrid, Spain; HIV Unit, Infectious Diseases Service, Hospital Clínic de Barcelona, Barcelona, Spain; CIBER de Enfermedades Infecciosas (CIBERINFEC), Instituto de Salud Carlos III, Madrid, Spain; Infectious Diseases Group, Department of Internal Medicine, Infanta Sofia University Hospital and Henares University Hospital Foundation for Biomedical Research and Innovation (FIIB HUIS HHEN), Madrid, Spain; Department of Medicine, Faculty of Medicine, Health and Sports, Universidad Europea, Madrid, Spain; Servicio de Medicina Interna, Hospital Universitario Príncipe de Asturias Alcalá de Henares, Madrid, Spain; HIV Unit, Hospital Universitari MutuaTerrassa, University of Barcelona, Barcelona, Spain; Department of Infectious Diseases, Hospital Universitario Ramón y Cajal, IRYCIS, Madrid, Spain; CIBER de Enfermedades Infecciosas (CIBERINFEC), Instituto de Salud Carlos III, Madrid, Spain; Department of Medicine, Universidad de Alcalá, Madrid, Spain; Department of Infectious Diseases, Hospital Universitario Ramón y Cajal, IRYCIS, Madrid, Spain; CIBER de Enfermedades Infecciosas (CIBERINFEC), Instituto de Salud Carlos III, Madrid, Spain; Department of Infectious Diseases, Hospital Universitario Ramón y Cajal, IRYCIS, Madrid, Spain; CIBER de Enfermedades Infecciosas (CIBERINFEC), Instituto de Salud Carlos III, Madrid, Spain

**Keywords:** antiretroviral therapy, HIV, inflammation, proteomics, two-drug regimens

## Abstract

**Background:**

While triple-drug regimens (3DR) have long been the standard of care for HIV infection, two-drug regimens (2DR), particularly dolutegravir/lamivudine (DTG/3TC), have emerged as viable first-line options. However, there is limited understanding of how baseline clinical profiles associated with regimen choice relate to underlying inflammatory states and long-term immune trajectories.

**Methods:**

We performed a retrospective observational study using data from the Spanish CoRIS cohort, including ART-naive individuals who initiated either DTG/3TC or bictegravir/emtricitabine/tenofovir alafenamide (BIC/F/TAF) between 2016 and 2023. We applied propensity score modeling to identify predictors of regimen choice. In a matched subset of participants with plasma samples at baseline and 24 months post-ART, we carried out longitudinal inflammatory profiling using the Olink Target 96 Inflammation panel. We then conducted differential expression and enrichment analyses and explored associations between clinical variables and proteomic changes over time.

**Results:**

Among 3145 participants (69.5% on BIC/F/TAF and 20.5% on DTG/3TC), those with higher baseline HIV-1 RNA and lower CD4+ T-cell counts were more likely to initiate BIC/F/TAF. In a matched subset (*n* = 174), 11 proteins were significantly overexpressed at baseline in the BIC/F/TAF group, suggesting a heightened inflammatory state. Both regimens led to significant downregulation of inflammatory markers over 2 years, though each displayed distinct proteins and functional pathways. Baseline viral load and CD4+ counts correlated with specific proteomic profiles and predicted longitudinal changes, particularly in the BIC/F/TAF group.

**Conclusions:**

Regimen selection was associated with baseline disease severity and inflammatory burden. Despite being used in patients with more advanced profiles, BIC/F/TAF effectively reduced systemic inflammation over 2 years. Both regimens attenuated inflammatory activity, though with distinct trajectories that may carry implications for immune recovery and long-term outcomes.

Antiretroviral therapy (ART) has transformed the clinical course of HIV-1 infection, turning it from a fatal disease into a chronic condition. Triple-drug regimens (3DR), typically combining two nucleoside reverse transcriptase inhibitors with a third agent, have long been the standard of care, providing robust viral suppression and a high genetic barrier to resistance [1–3]. However, concerns about long-term toxicity, drug–drug interactions, and patient burden have driven interest in regimen simplification. Two-drug regimens (2DR), especially dolutegravir plus lamivudine (DTG/3TC) [4], have emerged as viable alternatives for treatment-naive people living with HIV (PLHIV), demonstrating noninferiority in viral suppression compared to 3DR in large, randomized trials.

While clinical guidelines increasingly position 2DR as a first-line option [1, 2], the real-world factors influencing the choice between dual and triple regimens remain poorly defined. Variables such as viral load, CD4+ T-cell count, resistance profiles, comorbidities, and patient preference may inform clinical decisions [5, 6]. However, few studies have systematically investigated which patient characteristics predict initial ART choice in practice, especially in diverse healthcare settings. Moreover, even less is known about how these choices intersect with the underlying biological status of the patient, particularly regarding immune activation and systemic inflammation.

Persistent immune activation despite virologic suppression is a well-recognized driver of HIV-related comorbidities, including cardiovascular disease, cancer, and neurocognitive impairment, among others [7–12]. Prior studies suggest that different ART regimens may vary in their immunomodulatory effects, influencing levels of inflammatory biomarkers, immune cell activation, and even microbial translocation [13–18]. However, direct comparisons of the inflammatory trajectories associated with initial ART with 2DR versus 3DR over extended periods remain scarce and derived from switching studies [14, 19–22]. Understanding whether the choice of ART regimen can modulate the chronic inflammatory state is critical for optimizing long-term health outcomes in PLHIV, especially as life expectancy continues to improve.

In this study, we aimed to integrate clinical and basic science perspectives by combining predictive modeling of ART regimen selection with longitudinal inflammatory proteomic profiling. Using data from the Spanish CoRIS cohort, we first identify the sociodemographic and clinical factors that predict initiation of DTG/3TC versus bictegravir/emtricitabine/tenofovir alafenamide (BIC/F/TAF), two of the most widely prescribed first-line integrase strand transfer inhibitor (INSTI)-based regimens. We then employed targeted proteomic assays to characterize and compare the inflammatory protein profiles at baseline and after 2 years of ART. This combined approach allowed us to investigate whether clinical ART decisions reflect underlying biological heterogeneity and whether distinct immunologic trajectories emerge over time depending on treatment choice.

## METHODS

### Study Design and Participants

We conducted a retrospective observational study using data from the Spanish CoRIS cohort, a prospective, multicenter cohort that enrolls ART-naive adults living with HIV across 51 hospitals in Spain. CoRIS systematically collects demographic, clinical, and laboratory data, with standardized protocols applied across sites to ensure data comparability [23]. Participants provide informed consent for inclusion in the clinical database and for blood sample storage in a centralized biobank. Baseline plasma samples are obtained prior to ART initiation, and annual follow-up samples are collected thereafter. Internal quality controls are performed yearly, and an external audit evaluates 10% of the data every 2 years to ensure data integrity. For this analysis, we included all CoRIS enrollees who started either BIC/F/TAF or DTG/3TC as their first regimen. We focused on these regimens because they are currently the most widely prescribed first-line INSTI-based options: DTG/3TC represents the only dual therapy recommended for ART-naïve individuals, while BIC/FTC/TAF is the most frequently used 3DR.

### Clinical Predictors of Regimen Selection

To investigate the clinical factors associated with ART regimen choice, we calculated propensity scores (PS) using logistic regression models that included baseline variables: age, sex, race, HIV transmission category, educational level, HIV-1 RNA level (≥ or <100 000 copies/mL), CD4+ T-cell count (≥ or <200 cells/μL), body mass index category, estimated glomerular filtration rate (eGFR ≥ or <60 mL/min), corticosteroid or psychotropic medication use, diabetes, hypertension, dyslipidemia, and smoking status.

PS density distributions were plotted to visualize regimen selection patterns. We used logistic regression with backward stepwise variable selection to identify the strongest predictors of initiating BIC/F/TAF versus DTG/3TC.

### Proteomic Assays and Inflammatory Biomarker Profiling

To characterize inflammatory profiles, we selected a subset of participants with plasma samples available at both baseline (month 0, pre-ART) and 24 months (±6 months) post-ART initiation. Using 1:1 PS matching, we balanced participants between treatment groups based on age, sex, baseline CD4/CD8 ratio, and HIV-1 RNA level. The sample size for this analysis was limited by the restricted number of participants with sufficient follow-up in the DTG + 3TC group.

A total of 348 ethylenediaminetetraacetic acid (EDTA) plasma samples stored at −80°C were thawed, vortex-mixed, and prepared for proteomic analysis. Proteomic profiling was conducted using the Olink Target 96 Inflammation panel, a proximity extension assay-based platform that quantifies 92 inflammation-related proteins with high specificity and sensitivity. Samples passing Olink's internal quality control thresholds were retained for analysis (336 samples). Protein normalization was performed using Olink's standard intensity normalization method, except for S100A12 and Caspase-8, which were adjusted using interplate controls due to bimodal distributions [24]. Proteins were considered detected if measurable in >75% of samples across groups; those falling below this threshold were excluded from downstream analyses (8 proteins).

To minimize batch effects across the two analytical runs, we ensured balanced group representation within each run, and paired samples from a given participant were processed within the same batch. Normalized protein expression values, which provide relative protein quantification on a log2 scale, were used for statistical analysis [25].

### Statistical and Bioinformatic Analyses

We first conducted differential expression analyses of inflammatory proteins between treatment groups at baseline using Welch's two-sample *t*-test. We performed longitudinal within-group comparisons (baseline vs 24 months) using paired *t*-tests. To adjust for multiple comparisons, we applied false discovery rate (FDR) correction using the Benjamini–Hochberg method.

Functional analysis for the proteins studied was performed by enrichment analysis using the tool Metascape (http://metascape.org/) [26]. To ensure specificity to the experimental context, a custom background list comprising the 92 proteins quantified by the proteomic platform was used for all analyses. Enrichment analyses were conducted with a significance threshold set at *P* < .05. In addition to Metascape's default enrichment settings, all categories under “Functional Set” and “Pathway” were selected to provide a comprehensive assessment of biological processes, molecular functions, and signaling pathways associated with the differentially expressed proteins. Protein–protein interaction (PPI) network analysis was also performed within Metascape, and enriched functional terms inferred from PPI modules were reported when significant results were obtained.

Correlation analyses between significant clinical predictors (CD4+ count, viral load) and baseline protein expression were conducted using Spearman correlation coefficients. To evaluate whether baseline clinical predictors influenced the change in protein levels over time, we used linear regression models stratified by ART group. Viral load was log-transformed to meet normality assumptions. All variables were standardized prior to modeling to ensure that the resulting association estimates were comparable to correlation coefficients.

All statistical and bioinformatics analyses were performed using Stata v18.0 (StataCorp LP, College Station, TX, USA) and R software version 4.5.0 (R Foundation for Statistical Computing, Vienna, Austria), incorporating the appropriate packages (*haven, readxl, OlinkAnalyze, and tidyverse*).

## RESULTS

### Clinical Predictors of ART Regimen Selection

Between January 2016 and December 2023, 3145 PLHIV in the CoRIS cohort initiated ART with either BIC/F/TAF or DTG/3TC. Of these, 2187 (69.5%) received BIC/F/TAF and 958 (30.5%) received DTG/3TC. As shown in Table 1, both groups were comparable in most sociodemographic and clinical characteristics. However, we observed significant differences in baseline virologic and immunologic status: individuals initiating BIC/F/TAF were more likely to have a baseline HIV-1 RNA ≥100 000 copies/mL and a CD4+ T-cell count <200 cells/μL (*P* < .001 for both comparisons).

**Table 1.** Baseline Sociodemographic and Clinical Characteristics

	B/F/TAF (*n* = 2187)	DTG/3TC (*n* = 958)	*P*
Age, years, median (p25, p75)	46 (38, 56)	45 (37, 55)	.17
Male sex, *n* (%)	1843 (85)	788 (84)	.51
HIV transmission category, *n* (%)			.32
MSM	1318 (61)	564 (60)	
Heterosexual	604 (28)	273 (29)	
Injecting drug use	166 (8)	58 (6)	
Origin, *n* (%)			.004
Western Europe	1527 (71)	596 (64)	
Eastern Europe	36 (2)	14 (2)	
Africa	109 (5)	58 (6)	
Latin America	471 (22)	256 (28)	
Race, *n* (%)			.31
Black	74 (3)	39 (4)	
Other	2089 (97)	896 (98)	
Education level, *n* (%)			.83
No studies/primary	289 (13)	124 (13)	
Secondary	369 (17)	155 (16)	
College or higher	1169 (54)	509 (53)	
Nadir CD4, median (p25, p75)	325 (178, 498)	430 (313, 578)	<.001
CD4 <200 cell/μL, *n* (%)	581 (27)	47 (5)	<.001
CD4/CD8, median (p25, p75)	0.37 (0.20, 0.61)	0.51 (0.34, 0.75)	<0.001
HIV viral load >100 000 c/mL, median (p25, p75)	1067 (49)	280 (29)	<0.001
HIV viral load >500 000 c/mL, median (p25, p75)	419 (19)	87 (9)	<.001
Body mass index, median (p25, p75)	24 (21, 27)	24 (21, 27)	.29
Diabetes, *n* (%)	166 (8)	58 (6)	.12
Hypertension, *n* (%)	228 (10)	102 (11)	.85
Dyslipidemia, *n* (%)	413 (19)	188 (20)	.63
eGFR <60 mL/min/1.73 m^2^, *n* (%)	33 (2)	12 (1)	.59
Corticosteroids or psychotropics, *n* (%)	59 (3)	18 (2)	.17
Active smoking, *n* (%)	791 (47)	350 (43)	.082

**Figure 1.**
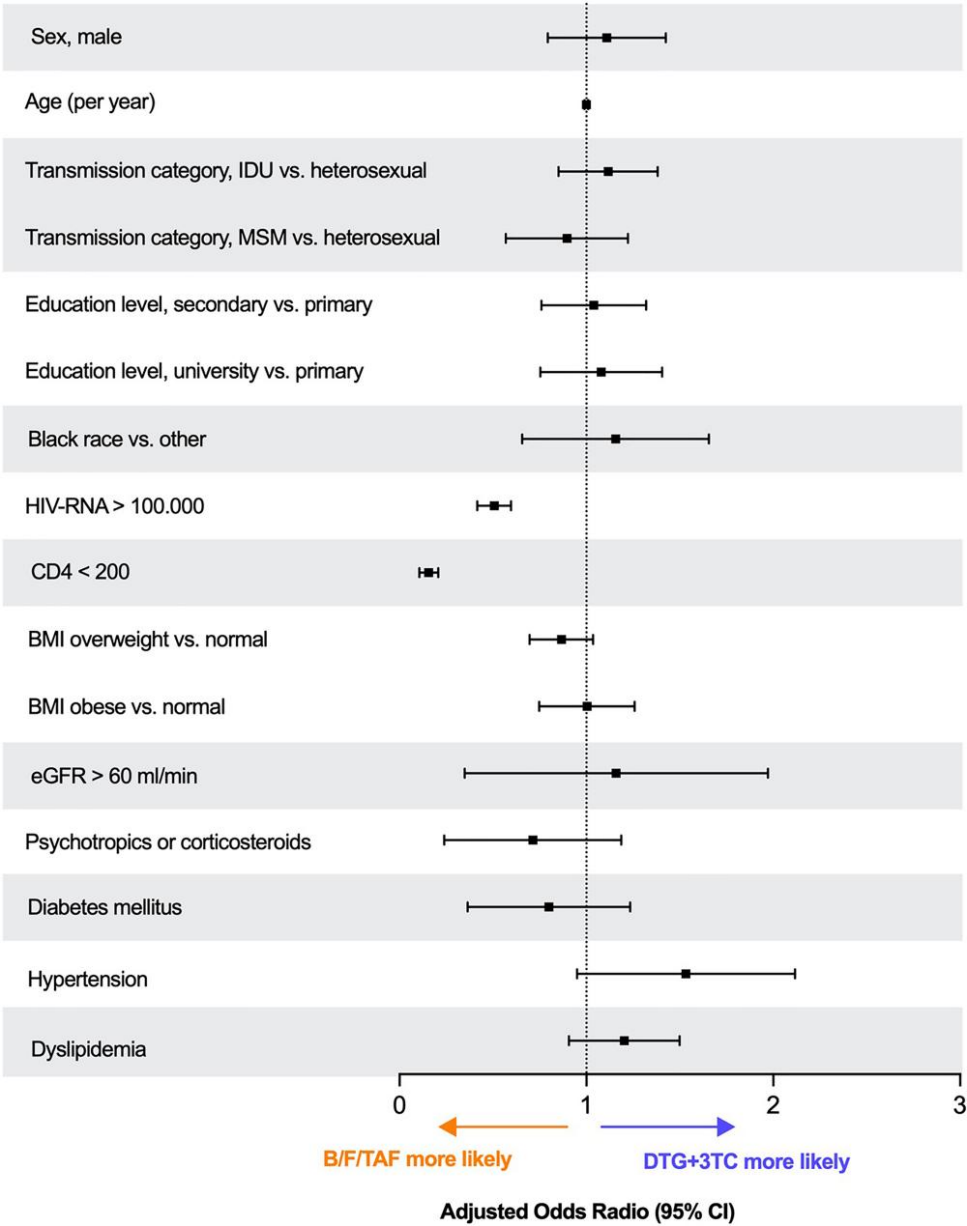
Clinical predictors of ART regimen selection. Forest plot showing adjusted odds ratios (ORs) and 95% confidence intervals (CIs) from a multivariable logistic regression model evaluating factors associated with the prescription of DTG/3TC versus BIC/F/TAF as initial ART regimen. ORs >1 indicate a higher likelihood of initiating DTG/3TC, while ORs <1 indicate a higher likelihood of initiating BIC/F/TAF.

PS density plots revealed distinct distributions between the two groups, suggesting the influence of baseline covariates on treatment choice (Supplementary Figure 1). The multivariable logistic regression model (Figure 1) confirmed that baseline viral load ≥100 000 copies/mL (odds ratio [OR] 0.49, 95% confidence interval [CI] 0.42–0.59) and CD4 + count <200 cells/μL (OR 0.15, 95% CI 0.11–0.21) were independently associated with a lower probability of receiving DTG/3TC. There was also a nonsignificant trend suggesting a higher probability of DTG/3TC initiation in individuals with dyslipidemia or hypertension. These findings indicate that, despite both regimens being guideline-endorsed first-line options, BIC/F/TAF is preferentially prescribed in patients with more advanced disease.

### Inflammatory Biomarker Profiles and Longitudinal Changes

We analyzed a PS-matched subset of 174 participants (88 on DTG/3TC and 86 on BIC/F/TAF) with available plasma samples at both baseline and at 24 months post-ART initiation. The median age in this subset was 37 years, with 92% male and 74% identifying as men who have sex with men (MSM). The median baseline CD4+ count was 380 cells/μL and the CD4/CD8 ratio was 0.5 (Supplementary Table 1).

**Figure 2.**
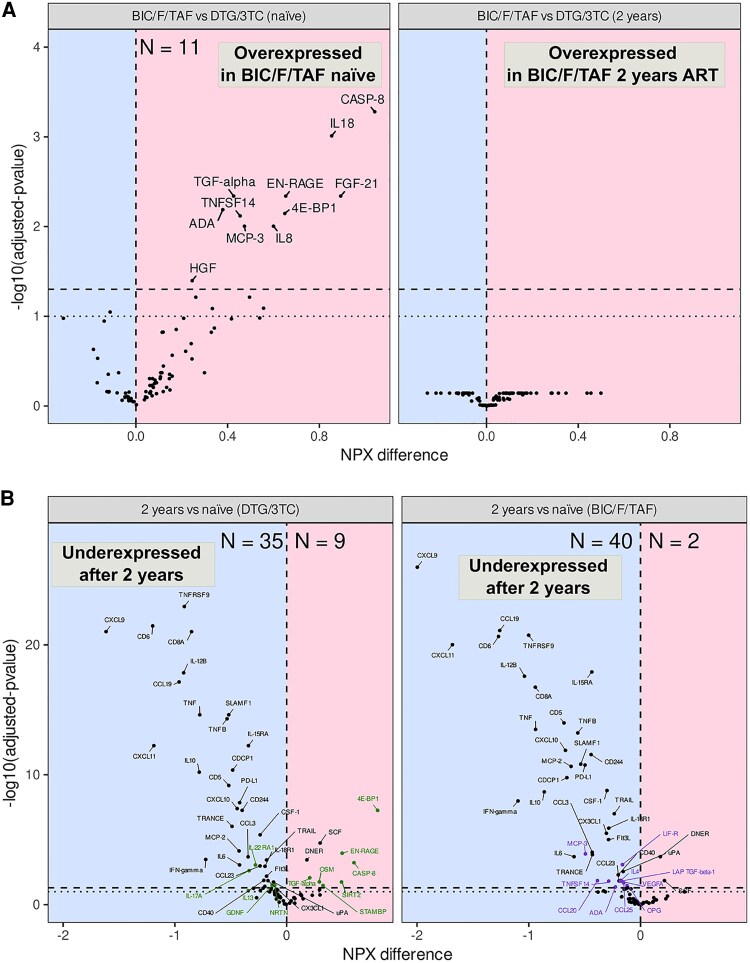
Differential expression analysis of proteins. (*A*) Differentially expressed proteins comparing treatment groups at baseline (month 0, left-hand side) and 2 y after ART initiation (month 24, right-hand side). Overexpressed proteins are shadowed in red and underexpressed proteins in blue. (*B*) Differentially expressed proteins comparing time points for the DTG/3TC (left-hand side) and BIC/F/TAF (right-hand side) groups. Overexpressed proteins are shadowed in red and underexpressed proteins in blue. Differentially expressed proteins only in DTG/3TC are highlighted in green. Differentially expressed proteins only in BIC/F/TAF are highlighted in purple.

At baseline, 11 inflammatory proteins were significantly overexpressed in the BIC/F/TAF group compared to the DTG/3TC group (Figure 2*A*). These included key cytokine mediators such as IL18 and CXCL8, chemokines, such as MCP-3 (CCL7), T-cell activators (TNSF14), tissue-repair (HGF), and metabolic regulators (FGF-21 and 4E-BP1). This suggests a heightened inflammatory state at ART initiation in patients receiving BIC/F/TAF. Importantly, after 2 years of ART, these differences were no longer detectable, reflecting effective immune modulation over time in both groups.

Longitudinal within-group comparisons (Figure 2*B* and Supplementary Table 2) showed substantial downregulation of inflammatory protein expression after 24 months in both regimens, particularly in chemokines and interleukins enriched for functions related to cell activation, leukocyte activation, interleukin signaling, viral defense, cell adhesion, and pathways such as natural killer cell-mediated cytotoxicity and Toll-like receptor signaling. Interestingly, each regimen exhibited unique protein expression profiles (green and purple color in Figure 2*B* and Supplementary Table 2). Specifically, 10 proteins were downregulated exclusively in the BIC/F/TAF group, including IL-4, TGF-b1, and some chemokines, and were enriched for functions related to lymphocyte homeostasis, leukocyte migration, and cell–cell adhesion. In contrast, DTG/3TC treatment resulted in the specific upregulation of 7 proteins including 4E-BP1, caspase-8, and S100-A12, and downregulation of 5 proteins, such as GDNF and IL-17A with enrichment analysis highlighting roles in mitotic cell cycle processes and positive regulation of the cell cycle. These findings suggest that, while both regimens suppress inflammation broadly, they differentially modulate pathways related to immune regulation and cellular proliferation.

### Correlation Between Clinical Predictors and Inflammatory Proteins

**Figure 3.**
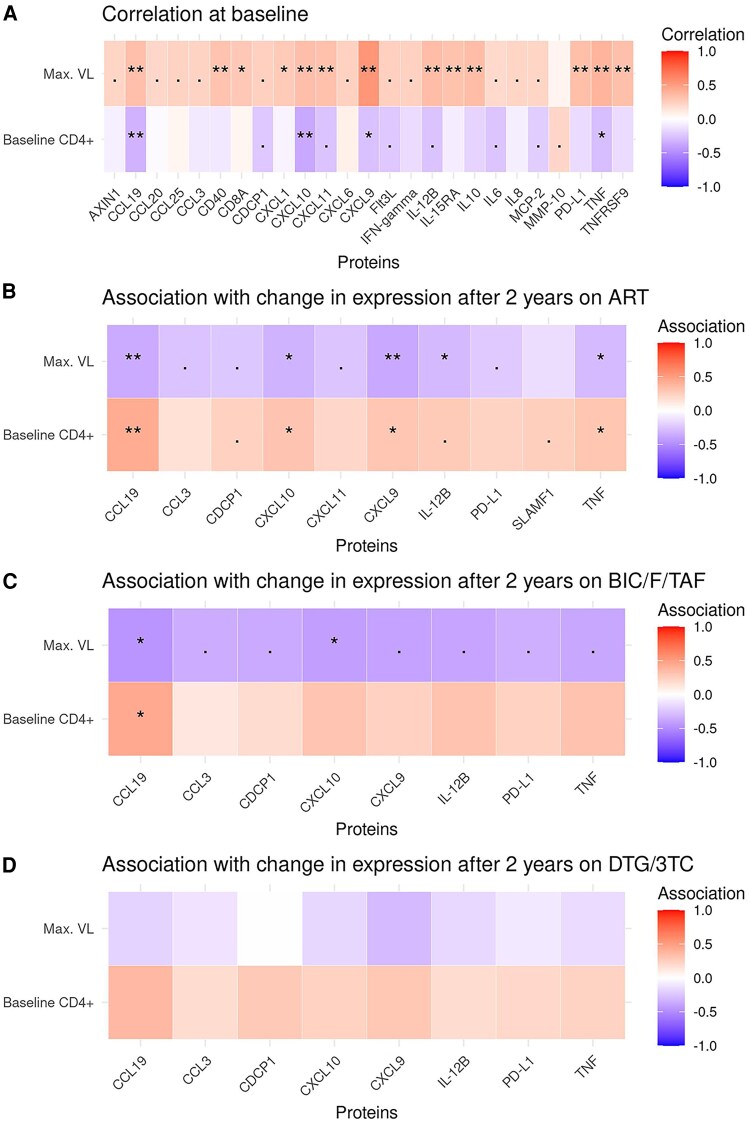
Associations between clinical variables and inflammatory protein expression. (*A*) Spearman correlation matrix between clinical variables [maximum viral load (VL) and baseline CD4+ counts] and the expression levels of 84 inflammatory proteins before ART initiation. (*B–D*) Associations between clinical variables (maximum VL and baseline CD4+ counts), and the change in protein expression after two years of ART, modeled using linear regression. Panel *B* includes all participants; panels *C* and *D* show stratified analyses for the BIC/F/TAF and DTG/3TC groups, respectively. Symbols indicate statistical significance based on adjusted *P*-values (adj. *P*): [**] = adj. *P* < .001, [*] = adj. *P* < .01, [.] = adj. *P* < .05, [] = adj. *P* ≥ .05 (not significant).

To explore whether clinical predictors of regimen choice reflected biological differences at baseline, we analyzed correlations between baseline CD4+ T-cell counts and maximum HIV-1 RNA (both significant predictors of ART choice) with the 84 inflammatory proteins baseline levels (Figure 3*A*). CD4+ count showed a significant negative correlation with 11 proteins, whereas maximum viral load correlated positively with 24 proteins, indicating a broad proinflammatory profile linked to uncontrolled viral replication. The strongest correlation was with CXCL9 (r = 0.54, adjusted *P* < .0001), which is a key interferon-inducible chemokine implicated in T-cell recruitment and systemic immune activation.

We then assessed whether these baseline clinical variables predicted longitudinal changes in protein expression over 24 months of ART (Figure 3*B*). Baseline CD4+ counts predicted the expression change of 7 proteins, while viral load predicted changes in 9. Changes in 6 proteins (CCL19, CDCP1, CXCL10, CXCL9, IL-12B, and TNF) were predicted by both clinical variables, but with opposite associations.

Stratified analysis by treatment group revealed that these associations were primarily observed in the BIC/F/TAF group: 8 protein changes were significantly linked to baseline viral load and 1 to CD4+ count (Figure 3*C*). In contrast, no significant associations were observed in the DTG/3TC group (Figure 3*D*).

## DISCUSSION

In this multicenter observational study, we integrated clinical modeling with targeted proteomic profiling to investigate factors driving antiretroviral regimen selection and their immunologic correlates in PLHIV. Combining real-world prescription data with longitudinal inflammation profiling, we demonstrated that clinical decisions about ART initiation not only reflect virologic and immunologic status, but are also associated with unmeasured factors related to inflammation.

Our predictive model confirmed that, in clinical practice, individuals with higher viral load and lower CD4+ T-cell counts were more likely to be prescribed BIC/F/TAF, while DTG/3TC tended to be initiated in patients with more favorable baseline profiles. This pattern is consistent with the historical caution reflected in international guidelines, which have either advised against DTG + 3TC in patients with advanced immunosuppression or high HIV viral load or highlighted the limited evidence available in such subgroups. In contrast to other guidelines such as those of EACS [1], DHHS [3], and IAS-USA [3], the 2025 update of the Spanish AIDS Study Group guidelines on ART for treatment-naive patients has removed such restrictions [27]. Future prospective studies will be necessary to assess whether clinical practice continues to differentiate the choice of regimen based on immunovirologic severity, even in the absence of formal recommendations. In parallel, the growing adoption of rapid ART initiation practices may also have shaped treatment patterns [28], although in our cohort we did not observe major differences in time to treatment initiation between the two groups.

Beyond clinical predictors, our proteomic analysis offers novel insights into the inflammatory profiles associated with ART choice. At baseline, individuals initiating BIC/F/TAF exhibited significantly higher levels of 11 inflammatory proteins, including chemokines (CXCL9, CXCL11) and T-cell activation markers (CD6), compared to those initiating DTG/3TC. This elevated inflammatory profile aligns with the more advanced immunologic status usually observed clinically at baseline and supports the notion that treatment choice is influenced by the disease severity, correlated with systemic inflammation. Notably, after 2 years of ART, these proteomic differences between regimens were no longer detectable, suggesting that both treatments effectively modulate the inflammatory milieu over time. This finding reinforces the concept that early immune activation can be modulated with sustained virologic suppression, regardless of regimen composition, consistent with prior reports on ART-induced immune recovery [29, 30]. However, we cannot rule out that additional factors related to regimen composition or host response may also contribute to shaping the inflammatory response. Furthermore, our study is the first to show that the two ART regimens differ in their effect on a wide panel of inflammatory markers. For example, the decrease of IL-4 after BIC/F/TAF treatment is interesting, since it has been associated with reduced chemokine expression (CCL20) [31], inflammatory pathways (IL-12p40, in mice) [32] and HIV inhibition through downregulation of CXCR4 expression [33]. However, the studies related with the effect of BIC/F/TAF mainly focus on CD4/CD8 counts and a few classical inflammatory cytokines. Similarly, cell cycle-related proteins such as 4E-BP1, caspase-8, or GDNF, had not been previously studied in the specific context of DTG/3TC treatment [34–36].

The observed correlations between baseline immunovirologic parameters and inflammatory protein expression illustrate the inflammatory consequences of classical prognostic markers. Baseline inflammation correlated more strongly with plasma HIV-1 viral load than with low CD4 counts. Maximum viral load positively correlated with 24 inflammatory proteins, most notably CXCL9, a strong interferon-inducible chemokine linked to systemic inflammation in HIV and described as a plasma biomarker in viremic patients [37–39], and TNF, IL-12B, which are related with acute infection [40, 41]. Baseline clinical variables also predicted the trajectory of several proteins over time—especially in the BIC/F/TAF group—supporting the idea that disease severity at ART initiation can shape immunologic recovery, and that regimen-specific effects may amplify or attenuate these trajectories. Again, although there is no direct evidence about the role of the treatment, some of these proteins, such as CCL19, CCL3, CXCL10, CXCL9, and TNF have been found overexpressed in noncontrollers [37].

While our data demonstrate substantial downregulation of inflammatory proteins in both treatment groups, we cannot determine whether this modulation represents complete normalization of the immune-inflammatory state. Although early initiation of ART is known to improve immune activation, no study has conclusively demonstrated that inflammation is completely reversed by virologic suppression alone [42]. Importantly, the finding that both treatment groups effectively modulate inflammation over time does not suggest that dual and triple regimens are interchangeable in all clinical contexts. While DTG/3TC demonstrated a similarly robust inflammatory downregulation over 2 years, it was used in patients with more favorable baseline profiles. This observation highlights the gap that has existed in the evidence regarding the safety and efficacy of dual regimens in populations with advanced HIV disease, such as those with CD4+ cell counts below 200 cells/μL or elevated baseline viremia, groups consistently underrepresented in clinical trials [4]. However, recent studies have evaluated the immunologic and clinical outcomes of DTG/3TC in these high-risk populations, with inconsistent results [43–45]. This evolving evidence has generated changes in some ART guidelines, such as the Spanish AIDS Study Group guidelines, which have lifted prior restrictions on DTG/3TC use in naive patients with advanced disease. In contrast, other international guidelines continue to maintain the recommendation against initiating DTG/3TC either in people with CD4 count <200 cells/μL [3] or with HIV-1 RNA > 500 000 copies/mL [1–3].

Our study has several strengths. Combining clinical data with high-resolution proteomic profiling provides a detailed understanding of how the evaluated ART regimens differentially affect inflammation. The use of a large, well-characterized national cohort enhances the relevance of the findings. Furthermore, the application of PS matching helped minimize baseline confounders between groups. However, several limitations must be acknowledged. A perfect balance was not achieved in important variables, such as CD4 count, although the differences were not clinically relevant. Nevertheless, to address this limitation, we performed correlation analysis by identifying proteins whose change correlates with baseline CD4. In addition, adherence data are not systematically collected within CoRIS, preventing us from assessing its potential influence on regimen selection. Key populations such as women, individuals from non-MSM transmission categories, and racially diverse groups were underrepresented, potentially affecting the generalizability of our findings. As the proteomic panel was broad, our analyses should be viewed as exploratory. We applied rigorous quality control measures and used FDR adjustment; nevertheless, validation in independent cohorts will be essential to confirm and contextualize our findings.

In conclusion, this integrated clinical and molecular analysis provides real-world evidence on the factors guiding ART selection and their association with systemic inflammation in PLHIV. Both DTG/3TC and BIC/FTC/TAF effectively reduced inflammatory biomarkers over 2 years, despite being prescribed to patient populations with different baseline profiles. Regimen choice was closely linked to baseline disease severity, with BIC/FTC/TAF more frequently initiated in individuals with more unfavorable immunological and inflammatory profiles. Nevertheless, despite differing baseline profiles, both strategies proved effective in reducing inflammation over 2 years. These findings emphasize the importance of personalized ART strategies that consider not only virologic efficacy but also immunologic context, particularly in populations at higher risk of persistent immune activation. Future studies should explore whether tailored treatment approaches based on inflammatory and immune profiles can further optimize long-term outcomes for PLHIV.

## Supplementary Material

ofaf626_Supplementary_Data

## Supplementary Data

Supplementary materials are available at *Open Forum Infectious Diseases* online. Consisting of data provided by the authors to benefit the reader, the posted materials are not copyedited and are the sole responsibility of the authors, so questions or comments should be addressed to the corresponding author.
