# Supplementary material for: Two or Three? Clinical and Proteomic Perspectives on Dolutegravir/Lamivudine Versus Bictegravir/Emtricitabine/Tenofovir Alafenamide as Initial HIV Treatment
Source: Open Forum Infect Dis. 2025 Oct 6;12(11):ofaf626. doi: 10.1093/ofid/ofaf626 (PMC12575079; doi:10.1093/ofid/ofaf626)
Supplement: ofaf626_Supplementary_Data [file ofaf626_supplementary_data.docx]

**Supplementary Material**

Two or three? Clinical and proteomic perspectives on dual versus triple antiretroviral therapy as initial HIV treatment.

*Claudio Díaz-García^1,2,3^, Sergio Serrano-Villar,^1,2^, Alejandro G. García-Ruiz de Morales^1,2^, Robert Güerri-Fernández^2,4^, Juncal Pérez-Somarriba^2,5^, Sonsoles Sánchez Palomino^2,6^, Inés Suárez-García^2,7^, Cristina Hernández Gutiérrez^8^, David Dalmau Juanola^9^, Santiago Moreno^1,2^, Elena Moreno^1,2^*, Javier Martínez-Sanz^1,2^*, on behalf of CoRIS*

**Co-senior authors, corresponding authors*

1. Department of Infectious Diseases, Hospital Universitario Ramón y Cajal, IRYCIS, Madrid,

Spain

2. CIBERINFEC, Instituto de Salud Carlos III, Madrid, Spain

3. Universidad de Alcalá, Department of Medicine, Madrid, Spain

4. Sección de Enfermedades Infecciosas, Hospital del Mar. Hospital del Mar Research Institute. Group for Research in Viral Infections (GRIV). Barcelona, Spain. Medicine and Life Sciences Department (MELIS). Univ Pompeu Fabra, Barcelona. Spain.

5. Unidad de enfermedades infecciosas, Hospital Clínico San Carlos, Madrid, Spain.

6. Hospital Clínic de Barcelona.

7. Infectious Diseases Group, Department of Internal Medicine, Infanta Sofia University Hospital, Infanta Sofia University Hospital and Henares University Hospital Foundation for Biomedical Research and Innovation (FIIB HUIS HHEN), Madrid, Spain. Department of Medicine. Faculty of Medicine, Health and Sports.

8. Servicio de Medicina Interna. Hospital Universitario Príncipe de Asturias Alcalá de Henares. Madrid.

9. HIV Unit. Hospital Universitari MutuaTerrassa. University of Barcelona, Barcelona, Spain

**Table of contents**

**Table S1**. Baseline and follow-up clinical characteristics of participants included in the proteomic substudy, stratified by ART regimen.

**Table S2.** Differentially expressed proteins in the two regimes after two years of treatment.

**Figure S1**. Distribution of propensity scores for individuals initiating BIC/FTC/TAF and DTG/3TC.

**Annex 1.** Centers and researchers involved in CoRIS.

**Table S1. Baseline and follow-up clinical characteristics of participants included in the proteomic substudy, stratified by ART regimen.**

|  | **BIC+FTC+TAF**  **(n = 86)** | **DTG+3TC**  **(n = 88)** | **p-value** |
| --- | --- | --- | --- |
| **Age, median (IQR)** | 37.3 (29.4, 46.3) | 35.2 (28.3, 42.3) | 0.33 |
| **Male sex at birth, n (%)** | 78 (91) | 83 (94) | 0.36 |
| **Transmission category, n (%)** |  |  | 0.38 |
| **MSM** | 60 (70) | 69 (78) |  |
| **Injecting drug use** | 0 (0) | 1 (1) |  |
| **Heterosexual** | 22 (26) | 15 (17) |  |
| **Highest education level, n (%)** | 4 (5) | 3 (3) | 0.078 |
| **Primary complete** | 15 (17) | 6 (7) |  |
| **Secondary complete** | 12 (14) | 16 (18) |  |
| **High school complete** | 28 (33) | 39 (44) |  |
| **University or higher** | 25 (29) | 28 (32) |  |
| **Country of origin, n (%)** |  |  | 0.019 |
| **Western Europe** | 50 (58) | 43 (49) |  |
| **Africa** | 7 (8) | 8 (9) |  |
| **Latin America** | 28 (33) | 32 (36) |  |
| **Virologic failure ever, n (%)*** | 1 (1) | 0 (0) | 0.31 |
| **Active smoking, n (%)** | 35 (42) | 28 (39) | 0.68 |
| **Nadir CD4, median (IQR)** | 353.5 (214.0, 470.0) | 407.0 (291.0, 555.0) | 0.024 |
| **Baseline CD4, median (IQR)** | 369.0 (230.0, 510.0) | 442.0 (314.0, 652.0) | 0.005 |
| **Baseline CD4/CD8, median (IQR)** | 0.5 (0.2, 0.6) | 0.5 (0.3, 0.7) | 0.009 |
| **Two-year CD4, median (IQR)** | 633.5 (448.0, 839.0) | 737.0 (533.0, 929.0) | 0.047 |
| **Two-year CD4/CD8, median (IQR)** | 0.8 (0.5, 1.1) | 0.8 (0.6, 1.1) | 0.34 |

** All matched participants maintained virologic suppression at 24 months. The single case of prior virologic failure was transient, with re-suppression achieved and no impact on proteomic analyses.*

**Table S2. Differentially expressed proteins in the two regimes after two years of treatment.**

| **Assay** | **UniProt** | **Protein name** | **DE in** | **log2FC** | **p-value** | **Adj. p-value** | **log2FC** | **p-value** | **Adj. p-value** | **METASCAPE-Enrichment Functions** |
| --- | --- | --- | --- | --- | --- | --- | --- | --- | --- | --- |
| ADA | P00813 | Adenosine deaminase | BIC | 0,07 | 3,01E-01 | 0,35 | -0,22 | 2,18E-02 | 0,04 | lymphocyte homeostasis regulation of leukocyte migration skeletal system development  PPI: negative regulation of cell development PPI: negative regulation of cell-cell adhesión |
| CCL20 | P78556 | C-C motif chemokine 20 | BIC | -0,30 | 3,02E-02 | 0,06 | -0,39 | 5,84E-03 | 0,01 |  |
| CCL25 | O15444 | C-C motif chemokine 25 | BIC | -0,11 | 8,56E-02 | 0,12 | -0,15 | 9,98E-03 | 0,02 |  |
| IL4 | P05112 | Interleukin-4 | BIC | -0,09 | 2,54E-01 | 0,30 | -0,18 | 5,87E-03 | 0,01 |  |
| LAP TGF-beta-1 | P01137 | Transforming growth factor beta-1 proprotein | BIC | -0,11 | 1,22E-01 | 0,17 | -0,18 | 7,13E-03 | 0,02 |  |
| LIF-R | P42702 | Leukemia inhibitory factor receptor | BIC | -0,09 | 3,80E-02 | 0,07 | -0,16 | 2,95E-04 | 0,00 |  |
| MCP-3 | P80098 | C-C motif chemokine 7 | BIC | -0,15 | 6,89E-02 | 0,10 | -0,49 | 3,81E-05 | 0,00 |  |
| OPG | O00300 | Tumor necrosis factor receptor superfamily member 11B | BIC | -0,10 | 4,27E-02 | 0,07 | -0,12 | 1,68E-02 | 0,03 |  |
| TNFSF14 | O43557 | Tumor necrosis factor ligand superfamily member 14 | BIC | -0,09 | 3,32E-01 | 0,37 | -0,28 | 6,96E-03 | 0,02 |  |
| VEGFA | P15692 | Vascular endothelial growth factor A, long form | BIC | -0,11 | 6,01E-02 | 0,09 | -0,17 | 6,96E-03 | 0,02 |  |
| CCL19 | Q99731 | C-C motif chemokine 19 | both | -0,96 | 5,14E-19 | 0,00 | -1,26 | 1,85E-23 | 0,00 | cell activation regulation of leukocyte activation HALLMARK INFLAMMATORY RESPONSE immune effector process regulation of interleukin-12 production HALLMARK IL2 STAT5 SIGNALING regulation of interleukin-1 production defense response to virus Cell adhesion molecules Natural killer cell mediated cytotoxicity regulation of macrophage migration Hematopoietic cell lineage blood coagulation cell recognition leukocyte migration involved in inflammatory response phosphatidylinositol 3-kinase/protein kinase B signal transduction PPI: regulation of interleukin-1 production  PPI: Toll-like receptor signaling pathway  PPI: Toll like receptor signaling |
| CCL23 | P55773 | C-C motif chemokine 23 | both | -0,24 | 3,84E-04 | 0,00 | -0,19 | 6,37E-03 | 0,01 |  |
| CCL3 | P10147 | C-C motif chemokine 3 | both | -0,35 | 6,00E-05 | 0,00 | -0,43 | 2,74E-05 | 0,00 |  |
| CD244 | Q9BZW8 | Natural killer cell receptor 2B4 | both | -0,40 | 1,12E-08 | 0,00 | -0,44 | 4,34E-13 | 0,00 |  |
| CD40 | P25942 | Tumor necrosis factor receptor superfamily member 5 | both | -0,20 | 5,80E-03 | 0,01 | -0,20 | 1,91E-03 | 0,01 |  |
| CD5 | P06127 | T-cell surface glycoprotein CD5 | both | -0,52 | 1,11E-10 | 0,00 | -0,68 | 1,09E-15 | 0,00 |  |
| CD6 | P30203 | T-cell differentiation antigen CD6 | both | -1,20 | 8,29E-24 | 0,00 | -1,27 | 1,07E-22 | 0,00 |  |
| CD8A | P01732 | T-cell surface glycoprotein CD8 alpha chain | both | -0,85 | 4,54E-23 | 0,00 | -0,94 | 1,71E-18 | 0,00 |  |
| CDCP1 | Q9H5V8 | CUB domain-containing protein 1 | both | -0,48 | 6,13E-12 | 0,00 | -0,66 | 3,38E-11 | 0,00 |  |
| CSF-1 | P09603 | Macrophage colony-stimulating factor 1 | both | -0,24 | 1,00E-06 | 0,00 | -0,30 | 3,50E-10 | 0,00 |  |
| CX3CL1 | P78423 | Fractalkine | both | -0,17 | 6,27E-03 | 0,01 | -0,31 | 8,81E-07 | 0,00 |  |
| CXCL10 | P02778 | C-X-C motif chemokine 10 | both | -0,44 | 7,63E-09 | 0,00 | -0,67 | 1,89E-13 | 0,00 |  |
| CXCL11 | O14625 | C-X-C motif chemokine 11 | both | -1,19 | 7,51E-14 | 0,00 | -1,68 | 5,72E-22 | 0,00 |  |
| CXCL9 | Q07325 | C-X-C motif chemokine 9 | both | -1,62 | 3,40E-23 | 0,00 | -2,00 | 1,24E-28 | 0,00 |  |
| DNER | Q8NFT8 | Delta and Notch-like epidermal growth factor-related receptor | both | 0,18 | 1,11E-04 | 0,00 | 0,18 | 6,97E-05 | 0,00 |  |
| Flt3L | P49771 | Fms-related tyrosine kinase 3 ligand | both | -0,18 | 2,60E-03 | 0,01 | -0,29 | 2,78E-06 | 0,00 |  |
| IFN-gamma | P01579 | Interferon gamma | both | -0,72 | 9,79E-05 | 0,00 | -1,10 | 2,47E-09 | 0,00 |  |
| IL-12B | P29460 | Interleukin-12 subunit beta | both | -0,92 | 8,42E-20 | 0,00 | -1,04 | 2,16E-19 | 0,00 |  |
| IL-15RA | Q13261 | Interleukin-15 receptor subunit alpha | both | -0,34 | 6,84E-14 | 0,00 | -0,44 | 8,67E-20 | 0,00 |  |
| IL-18R1 | Q13478 | Interleukin-18 receptor 1 | both | -0,19 | 4,26E-04 | 0,00 | -0,28 | 3,37E-07 | 0,00 |  |
| IL10 | P22301 | Interleukin-10 | both | -0,78 | 9,75E-12 | 0,00 | -0,86 | 4,69E-10 | 0,00 |  |
| IL6 | P05231 | Interleukin-6 | both | -0,42 | 3,04E-04 | 0,00 | -0,60 | 7,07E-05 | 0,00 |  |
| MCP-2 | P80075 | C-C motif chemokine 8 | both | -0,43 | 1,92E-05 | 0,00 | -0,62 | 4,30E-12 | 0,00 |  |
| PD-L1 | Q9NZQ7 | Programmed cell death 1 ligand 1 | both | -0,42 | 2,49E-09 | 0,00 | -0,50 | 3,17E-12 | 0,00 |  |
| SCF | P21583 | Kit ligand | both | 0,30 | 4,51E-06 | 0,00 | 0,21 | 5,51E-03 | 0,01 |  |
| SLAMF1 | Q13291 | Signaling lymphocytic activation molecule | both | -0,52 | 2,32E-16 | 0,00 | -0,53 | 2,55E-12 | 0,00 |  |
| TNF | P01375 | Tumor necrosis factor | both | -0,78 | 2,03E-16 | 0,00 | -0,94 | 3,83E-15 | 0,00 |  |
| TNFB | P01374 | Lymphotoxin-alpha | both | -0,53 | 5,26E-16 | 0,00 | -0,56 | 7,81E-15 | 0,00 |  |
| TNFRSF9 | Q07011 | Tumor necrosis factor receptor superfamily member 9 | both | -0,92 | 1,34E-25 | 0,00 | -1,00 | 6,50E-23 | 0,00 |  |
| TRAIL | P50591 | Tumor necrosis factor ligand superfamily member 10 | both | -0,18 | 1,17E-04 | 0,00 | -0,23 | 2,51E-08 | 0,00 |  |
| TRANCE | O14788 | Tumor necrosis factor ligand superfamily member 11 | both | -0,49 | 2,13E-07 | 0,00 | -0,43 | 4,53E-05 | 0,00 |  |
| uPA | P00749 | Urokinase-type plasminogen activator | both | -0,12 | 8,65E-03 | 0,02 | -0,16 | 1,04E-03 | 0,00 |  |
| 4E-BP1 | Q13541 | Eukaryotic translation initiation factor 4E-binding protein 1 | DTG | 0,81 | 1,18E-08 | 0,00 | 0,24 | 1,36E-01 | 0,22 | mitotic cell cycle process positive regulation of cell cycle |
| CASP-8 | Q14790 | Caspase-8 | DTG | 0,60 | 1,97E-04 | 0,00 | -0,05 | 8,17E-01 | 0,89 |  |
| EN-RAGE | P80511 | Protein S100-A12 | DTG | 0,50 | 3,01E-05 | 0,00 | 0,00 | 9,89E-01 | 0,99 |  |
| GDNF | P39905 | Glial cell line-derived neurotrophic factor | DTG | -0,13 | 1,52E-02 | 0,03 | -0,03 | 5,81E-01 | 0,68 |  |
| IL-17A | Q16552 | Interleukin-17A | DTG | -0,34 | 9,50E-04 | 0,00 | -0,17 | 1,54E-01 | 0,24 |  |
| IL-22 RA1 | Q8N6P7 | Interleukin-22 receptor subunit alpha-1 | DTG | -0,28 | 3,11E-04 | 0,00 | -0,09 | 2,37E-01 | 0,35 |  |
| IL13 | P35225 | Interleukin-13 | DTG | -0,20 | 2,06E-02 | 0,04 | -0,17 | 1,63E-01 | 0,25 |  |
| NRTN | Q99748 | Neurturin | DTG | -0,11 | 1,61E-02 | 0,03 | -0,02 | 7,07E-01 | 0,78 |  |
| OSM | P13725 | Oncostatin-M | DTG | 0,29 | 7,93E-03 | 0,02 | -0,08 | 5,41E-01 | 0,65 |  |
| SIRT2 | Q8IXJ6 | NAD-dependent protein deacetylase sirtuin-2 | DTG | 0,49 | 8,29E-03 | 0,02 | 0,23 | 2,41E-01 | 0,35 |  |
| STAMBP | O95630 | STAM-binding protein | DTG | 0,33 | 1,71E-02 | 0,03 | 0,14 | 3,14E-01 | 0,43 |  |
| TGF-alpha | P01135 | Protransforming growth factor alpha | DTG | 0,21 | 3,56E-03 | 0,01 | -0,02 | 8,68E-01 | 0,92 |  |
| ARTN | Q5T4W7 | Artemin | none | -0,03 | 4,69E-01 | 0,51 | -0,05 | 3,40E-01 | 0,46 |  |
| AXIN1 | O15169 | Axin-1 | none | 0,07 | 7,26E-01 | 0,77 | 0,08 | 6,84E-01 | 0,77 |  |
| CCL11 | P51671 | Eotaxin | none | 0,07 | 2,46E-01 | 0,29 | -0,10 | 9,83E-02 | 0,16 |  |
| CCL28 | Q9NRJ3 | C-C motif chemokine 28 | none | -0,08 | 1,12E-01 | 0,16 | -0,12 | 2,59E-02 | 0,05 |  |
| CCL4 | P13236 | C-C motif chemokine 4 | none | 0,13 | 1,13E-01 | 0,16 | 0,12 | 1,90E-01 | 0,29 |  |
| CST5 | P28325 | Cystatin-D | none | 0,04 | 4,57E-01 | 0,51 | -0,06 | 2,92E-01 | 0,41 |  |
| CXCL1 | P09341 | Growth-regulated alpha protein | none | -0,34 | 5,43E-02 | 0,08 | 0,01 | 9,68E-01 | 0,98 |  |
| CXCL5 | P42830 | C-X-C motif chemokine 5 | none | -0,27 | 2,46E-01 | 0,29 | 0,39 | 5,95E-02 | 0,10 |  |
| CXCL6 | P80162 | C-X-C motif chemokine 6 | none | -0,24 | 5,05E-02 | 0,08 | -0,01 | 8,88E-01 | 0,92 |  |
| FGF-19 | O95750 | Fibroblast growth factor 19 | none | -0,07 | 5,89E-01 | 0,63 | 0,12 | 4,34E-01 | 0,56 |  |
| FGF-21 | Q9NSA1 | Fibroblast growth factor 21 | none | 0,15 | 3,10E-01 | 0,35 | -0,31 | 5,95E-02 | 0,10 |  |
| FGF-23 | Q9GZV9 | Fibroblast growth factor 23 | none | -0,06 | 2,41E-01 | 0,29 | 0,03 | 6,09E-01 | 0,70 |  |
| HGF | P14210 | Hepatocyte growth factor | none | 0,06 | 2,36E-01 | 0,29 | -0,06 | 3,97E-01 | 0,52 |  |
| IL-10RB | Q08334 | Interleukin-10 receptor subunit beta | none | -0,06 | 1,87E-01 | 0,24 | -0,08 | 3,22E-02 | 0,06 |  |
| IL-17C | Q9P0M4 | Interleukin-17C | none | -0,01 | 8,84E-01 | 0,89 | -0,06 | 5,53E-01 | 0,65 |  |
| IL-2RB | P14784 | Interleukin-2 receptor subunit beta | none | -0,08 | 4,56E-02 | 0,08 | -0,03 | 4,44E-01 | 0,57 |  |
| IL18 | Q14116 | Interleukin-18 | none | 0,23 | 1,42E-01 | 0,19 | -0,38 | 6,33E-02 | 0,11 |  |
| IL2 | P60568 | Interleukin-2 | none | -0,09 | 5,41E-02 | 0,08 | 0,01 | 8,61E-01 | 0,92 |  |
| IL5 | P05113 | Interleukin-5 | none | -0,21 | 2,87E-02 | 0,05 | -0,10 | 4,74E-01 | 0,59 |  |
| IL7 | P13232 | Interleukin-7 | none | -0,14 | 3,77E-02 | 0,07 | -0,06 | 2,36E-01 | 0,35 |  |
| IL8 | P10145 | Interleukin-8 | none | 0,29 | 4,23E-02 | 0,07 | -0,33 | 4,83E-02 | 0,09 |  |
| LIF | P15018 | Leukemia inhibitory factor | none | 0,00 | 9,44E-01 | 0,94 | -0,13 | 3,12E-02 | 0,06 |  |
| MCP-1 | P13500 | C-C motif chemokine 2 | none | 0,03 | 7,35E-01 | 0,77 | -0,15 | 3,63E-02 | 0,07 |  |
| MCP-4 | Q99616 | C-C motif chemokine 13 | none | 0,13 | 1,53E-01 | 0,20 | 0,01 | 8,91E-01 | 0,92 |  |
| MMP-1 | P03956 | Interstitial collagenase | none | -0,02 | 8,45E-01 | 0,88 | -0,11 | 2,54E-01 | 0,36 |  |
| MMP-10 | P09238 | Stromelysin-2 | none | -0,01 | 8,84E-01 | 0,89 | -0,05 | 3,92E-01 | 0,52 |  |
| NT-3 | P20783 | Neurotrophin-3 | none | -0,11 | 8,19E-02 | 0,12 | 0,03 | 6,86E-01 | 0,77 |  |
| ST1A1 | P50225 | Sulfotransferase 1A1 | none | 0,30 | 1,30E-01 | 0,18 | 0,11 | 5,50E-01 | 0,65 |  |
| TSLP | Q969D9 | Thymic stromal lymphopoietin | none | -0,08 | 1,34E-01 | 0,18 | 0,06 | 5,00E-01 | 0,62 |  |
| TWEAK | O43508 | Tumor necrosis factor ligand superfamily member 12 | none | -0,07 | 1,89E-01 | 0,24 | 0,00 | 9,60E-01 | 0,98 |  |

Differentially expressed proteins in the two regimes after two years of treatment. Proteins (Olink assay) have been ordered according to the following criteria: Differentially Expressed only in BIC/F/TAF (purple BIC), Differentially Expressed only in DTG/3TC (green DTG), Differentially Expressed in both (white, both) and non-differentially expressed (grey, none).

**Figure S1**


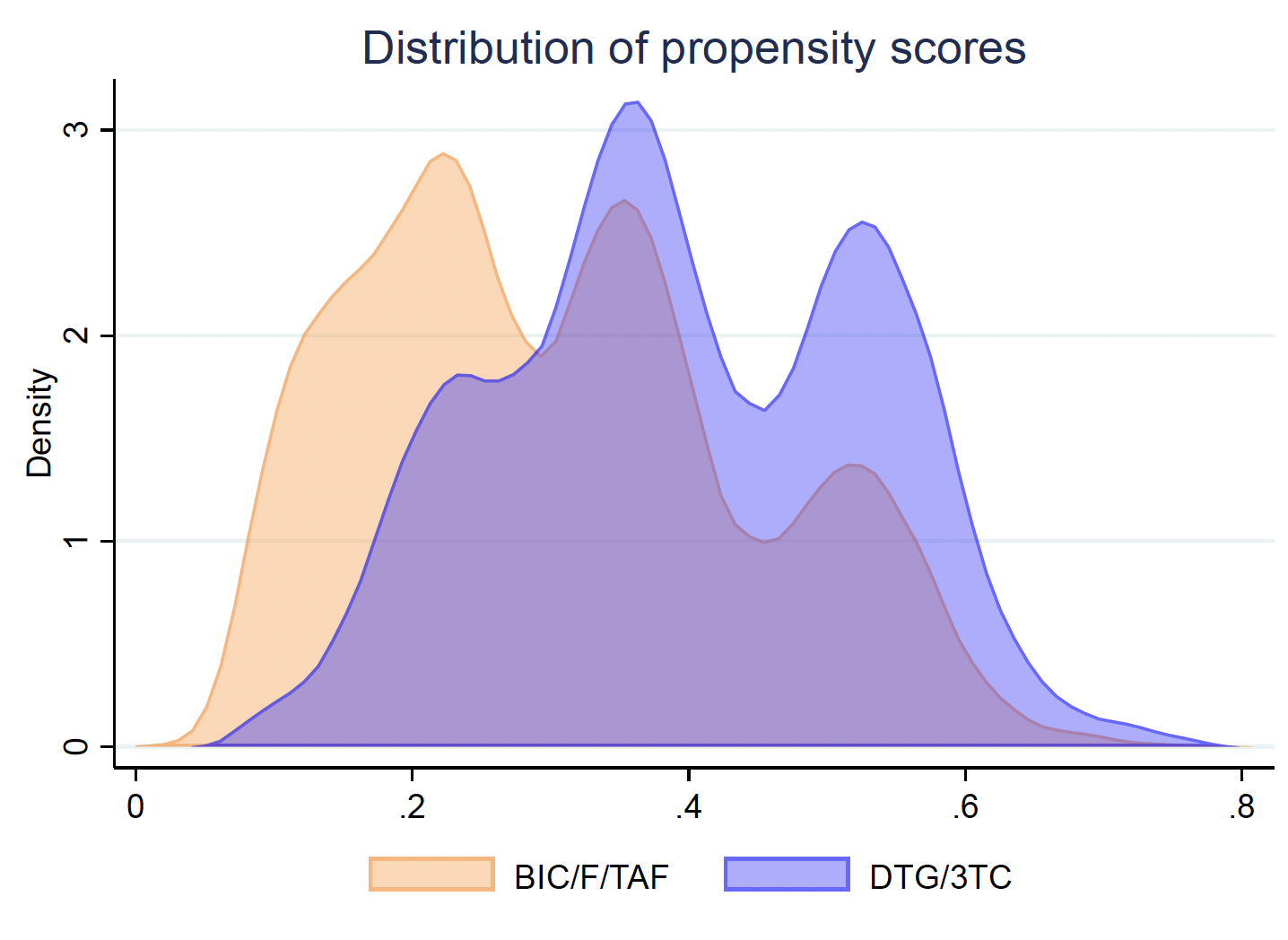


**Figure S1. Distribution of propensity scores for individuals initiating BIC/FTC/TAF and DTG/3TC.** Density plot showing the distribution of propensity scores calculated for each participant based on baseline sociodemographic and clinical covariates. Propensity scores reflect the estimated probability of initiating either BIC/F/TAF (orange) or DTG/3TC (blue) using multivariable logistic regression models. The figure illustrates partial overlap between groups and highlights systematic differences in the baseline likelihood of receiving each regimen.

**Annex 1.** **CENTERS AND RESEARCHERS INVOLVED IN CoRIS**

***CoRIS Executive committee:***

Santiago Moreno, Inma Jarrín, David Dalmau, M Luisa Navarro, Federico Garcia, Eva Poveda, Jose Antonio Iribarren, Félix Gutiérrez, Francesc Vidal, Juan Berenguer, Juan González.

***Centres and investigators involved in CoRIS cohort are listed below:***

**CoRIS Coordination Unit**

Inma Jarrín, Cristina Moreno, Marta Rava, Rebeca Izquierdo, Cristina Marco-Sánchez, Teresa Gómez-García.

**BioBanK HIV Hospital General Universitario Gregorio Marañón**

José Luis Jiménez.

**Hospital General Universitario Dr Balmis (Alicante)**

Sergio Reus, Irene Portilla, Esperanza Merino, Gema García, José Sánchez-Payá, Juan Carlos Rodríguez, Livia Giner, Joaquín Portilla, Vicente Boix, Diego Torrus, Julia Portilla-Tamarit, Héctor Pinargote.

**Hospital Universitario de Canarias (San Cristóbal de la Laguna)**

María Remedios Alemán, Nereyda Tosco-García, Ana López Lirola, Dácil García, Felicitas Díaz-Flores, María del Mar Alonso, Ricardo Pelazas, María Inmaculada Hernández, Lucia Romero-Acevedo, Abraham Bethencourt-Padilla, Daniel Rodríguez-Díaz, Ana María Godoy-Reyes.

**Hospital Universitario Central de Asturias (Oviedo)**

Víctor Asensi, Rebeca Cabo Magadan, Lorena Fernández, Javier Díaz-Arias

**Hospital Universitario 12 de Octubre (Madrid)**

Federico Pulido, Rafael Rubio, M Asunción Hernando, Otilia Bisbal, David Rial-Crestelo, María de Lagarde, Laura Bermejo, Mireia Santacreu, Juan Martín Torres, Belén Sánchez-López.

**Servicio de Enfermedades Infecciosas. Hospital Universitario Donostia. Instituto de Investigación BioDonostia (Donostia-San Sebastián)**

José Antonio Iribarren, Xabier Kortajarena, Claudia Nevado Pavón, Xabier Camino, Miguel Ángel Goenaga, M Jesús Bustinduy, Harkaitz Azkune, Maialen Ibarguren, Ignacio Álvarez-Rodriguez, Leire Gil-Alonso, Francisco Carmona-Torre, Ana Bayona Carlos, Maialen Lekuona Sanz.

**Hospital General Universitario De Elche (Elche)**

Félix Gutiérrez, Catalina Robledano, Mar Masiá, Sergio Padilla, Rafael Pascual, Marta Fernández, Antonio Galiana, José Alberto García, Xavier Barber, Javier García Abellán, Guillermo Telenti, Ángela Botella, Paula Mascarell, Lidia García-Sánchez, Nuria Ena, Leandro López, Jennifer Vallejo, Nieves Gonzalo-Jiménez, Montserrat Ruiz, Christian Ledesma, Santiago López, María Espinosa-Pérez, Ana Quiles, María del Mar Alcalde-Encinas, José García-García, Rosario Hernández-Ros, José Carlos Escribano, Marouane Menchi-Elanci, María del Mar García Navarro.

**Hospital General Universitario Gregorio Marañón (Madrid)**

Cristina Diez, Isabel Gutiérrez, Juan Berenguer, Margarita Ramírez, Teresa Aldamiz-Echevarría, Francisco Tejerina, Leire Pérez-Latorre, Chiara Fanciulli, Saray Corral.

**Hospital Universitari de Tarragona Joan XXIII (Tarragona)**

Joaquim Peraire, Anna Rull, Anna Martí, Consuelo Viladés, Beatriz Villar, Lluïsa Guillem, Silvia Chafino, Marina Flores, Francesc Vidal.

**Hospital Universitario y Politécnico de La Fe (Valencia)**

Marta Montero-Alonso, María Tasias-Pitarch, Eva Calabuig-Muñoz, Miguel Salavert-Lletí, Juan Fernández-Navarro, Rosa Blanes-Hernández, Jennifer Sánchez-Guevara.

**Hospital Universitario La Paz/IdiPAZ (Madrid)**

Juan González-García, José Ignacio Bernardino, Ana Delgado-Hierro, José Ramón Arribas, Víctor Arribas, Juan Miguel Castro, Luis Escosa, Iker Falces, Pedro Herranz-Pinto, Alicia González-Baeza, María Luz Martín-Carbonero, Rafael Micán, Rocío Montejano, María Luisa Montes, Luis Ramos-Ruperto, Berta Rodés Soldevila, Talia Sainz, Elena Sendagorta, Carmen Busca, Joanna Cano-Smith, Rosa de Miguel, María del Mar Arcos-Rueda, Alejandro de Gea-Grela, Nerea Iniesta-Arandia, Alejandro Díez-Vidal, María Jesús Roldán-Cabrales, Carlos Oñoro-López, Jara Llenas García, Laura Lucía Checa Daimiel.

**Hospital Universitari Mutua Terrassa (Terrassa)**

David Dalmau, Marina Martinez, Angels Jaén, Mireia Cairó, Javier Martinez-Lacasa, Roser Font, Laura Gisbert.

**Hospital Universitario de La Princesa (Madrid)**

Ignacio de los Santos, Alejandro de los Santos, Lucio García-Fraile, Enrique Martín-Gayo, Ildefonso Sánchez-Cerrillo, Ángela Gutiérrez, Carmen Sáez, Ana Barrios Blandino, Azucena Bautista, Marianela Ciudad, María Aguilera García, Violeta Sampériz Rubio, Javier Pérez Serrano, Isabel Belmonte Martín de Santa Olalla.

**Hospital Universitario Ramón y Cajal (Madrid)**

Santiago Moreno, Santos del Campo, José Luis Casado, Fernando Dronda, Ana Moreno, María Jesús Pérez-Elías, Sergio Serrano-Villar, María Jesús Vivancos-Gallego, Javier Martínez-Sanz, Alejandro Vallejo Tiller, Matilde Sánchez-Conde, José Antonio Pérez-Molina, José Manuel Hermida, Erick De La Torre Tarazona, Elena Moreno del Olmo, Laura Martín Pedraza, Claudio Díaz García, Jorge Díaz Álvarez, Alejandro García-García, Raquel Ron-González, Sergio Calderón Vicente, Roser Navarro Soler, Sara Saiz-Baggetto, Ana del Amo-de Palacios, Laura Luna, Miguel Antón Ámez Segovia, Sara Martín Colmenarejo, Cristina Chica.

**Hospital General Universitario Reina Sofía (Murcia)**

Enrique Bernal, María Dolores Hernández, Antonia Alcaraz, Joaquín Bravo, Ángeles Muñoz Pérez, Cristina Tomás Jimenez, Salvador Valero Cifuentes, Eva García-Villalba, Román González Hipólito, Elena Guijarro-Westermeyer, Rodrigo Martínez-Rodríguez, José Miguel Gómez Verdú.

**Hospital Universitario Clínico San Cecilio (Granada)**

Federico García, Clara Martínez, Maite Laperal, Leopoldo Muñoz Medina, Marta Álvarez-Estevez, Natalia Chueca-Porcuna, David Vinuesa-García, Adolfo de Salazar-González, Ana Fuentes-López, Emilio Guirao, Andrés Ruiz-Sancho, Francisco Anguita, Naya Faro, Lucia Chaves, Marta Illescas, Paloma Muñoz-Baez, Lucía Pérez, Ana Alberola Romano, Alberto Vazquez Blanquiño, Lucía Guillén-Zafra, Javier Martínez de Victoria-Carazo.

**Centro Sanitario Sandoval (Madrid)**

Jorge Del Romero-Guerrero, Montserrat Raposo, Teresa Puerta-López, Mar Vera, Juan Ballesteros, Begoña Baza, Laura Dans Villán, Ruben Linares Navarro, Ines Armenteros Yeguas, Eva Orviz-García, Santiago Fernández Castelao.

**Hospital Universitario Son Espases (Palma de Mallorca)**

Melchor Riera, Antonio Vanrell, María Peñaranda, Mª Angels Ribas, Antoni A. Campins, Mercedes Garcia-Gasalla, Francisco J Fanjul, Javier Murillas-Angoiti, Luisa Martin-Pena, Francisca Artigues, Sophia Pinecki, Adrián Ferre.

**Hospital Universitario Virgen de la Victoria (Málaga)**

Jesús Santos, María López-Jódar, Cristina Gómez-Ayerbe, Isabel Viciana, Rosario Palacios.

**Hospital Universitario Virgen del Rocío (Sevilla)**

Luis Fernando López-Cortés, Silvia Llaves-Flores, Inmaculada Rivas Jeremías, Nuria Espinosa, Cristina Roca-Oporto, Marta Herreros-Romero, César Sotomayor de la Piedra, Abraham Saborido Alconchel, Manuel Francisco Liroa, Jesús Fernández Plaza.

**Hospital Universitario de Bellvitge (Hospitalet de Llobregat)**

Juan Manuel Tiraboschi, Arkaitz Imaz, María Saumoy, Analuz Fernandez, Jaime Vega Costa, Daniel Medina Gamito.

**Hospital Costa del Sol (Marbella)**

Julián Olalla, Javier Pérez, Alfonso del Arco, Javier de la Torre, Francisca Ruiz.

**Hospital General Universitario Santa Lucía (Cartagena)**

Onofre Juan Martínez, Lorena Martinez, Francisco Jesús Vera, Josefina García, Begoña Alcaraz, Sergio Guillén Martínez, Patricia Carles García.

**Complejo Hospitalario Universitario a Coruña (CHUAC) (A Coruña)**

Álvaro Mena, Berta Pernas, Pilar Vázquez, Soledad López, Brais Castelo.

**Hospital Universitario Virgen de la Arrixaca (El Palmar)**

Carlos Galera, Marian Fernández, Helena Albendin, Antonia Castillo, Asunción Iborra, Antonio Moreno, M Angustias Merlos, Almudena Ortuño.

**Hospital Universitario Infanta Sofía (San Sebastián de los Reyes)**

Inés Suarez-García, Eduardo Malmierca, Patricia González-Ruano, M Pilar Ruiz, Luz Balsalobre, Ángela Somodevilla, Rebeca Fuerte Martínez.

**Hospital Clínico San Carlos (Madrid)**

Vicente Estrada, Nieves Sanz, Noemí Cabello-Clotet, María José Núñez, Ana Muñoz, Juncal Pérez-Somarriba, Reynaldo Homen, Rafael Rubio-Martín, Susana Olmedo, Julia Barrado.

**Hospital Universitario Príncipe de Asturias (Alcalá de Henares)**

José Sanz, Cristina Hernández-Gutiérrez, María Novella-Mena.

**Hospital Clínico Universitario de Valencia (Valencia)**

María José Galindo, Sandra Pérez Gómez, Ana Ferrer.

**Hospital Reina Sofía (Córdoba)**

Antonio Rivero-Román, Laura Ruiz-Torres, Antonio Rivero-Juárez, Pedro López-López, Mario Frias-Casas, Ángela Camacho, Ignacio Pérez, Diana Corona, Javier Manuel Caballero, Marina Gallo Marín, María Casares, Lucía Ríos-Muñoz, Claudia Ferreira-Tata, María Carrasquilla.

**Hospital Universitario Severo Ochoa (Leganés)**

Rafael Rodríguez-Rosado Martinez-Echevarría.

**Hospital Universitario Virgen de Valme (Sevilla)**

Juan Macías Sánchez, Pilar Rincón, Luis Miguel Real, Anaïs Corma, Jésica Martín-Carmona.

**Hospital Álvaro Cunqueiro (Vigo)**

Eva Poveda, Alexandre Pérez, Luis Morano, Celia Miralles, Antonio Ocampo, Jacobo Alonso, Inés Martínez, Aida López-López.

**Hospital Universitario Marqués de Valdecilla**

M Carmen Fariñas Álvarez, Claudia González Rico, Noelia Ruiz Alonso, Carlos Armiñanzas Castillo, Francisco Arnaiz de las Revillas Almajano, Manuel Gutiérrez Cuadra, Raúl Parra Fariñas, Paula Runza Buznego, Aitziber Illaro Uranga.

**Hospital Clínic de Barcelona**

José Luis Blanco Arévalo, Pilar Callau Cabrera, Josep Mallolas Masferrer, José Alcamí Pertejo, Sonsoles Sánchez-Palomino, Núria Climent, Ana González-Cordón, Montse Laguno Centeno, María Martínez-Rebollar, Juan Ambrosioni, Berta Torres, Lorena de la Mora, Alexy Inciarte, Esteban Martínez, José María Miró, Abiu Sempere, Julia Calvo, Iván Chivite, David García, Alberto Foncillas, Daniela Malano, Montse Plana, Roger Llobet, Estela Solbes, Octavi Roman, Rona Sagarra, Vanesa Guilera, Gemma Olmeda, Paula Arreba, María José Rodríguez, Emma Fernández, Ana Rodríguez, Alba ortega, Sergi Anguera, Raquel Aguiló, Laura Novell.

**Centro Nacional de Microbiología**

Luis Miguel Bedoya Del Olmo, Manuela Beltran Vicente, Mercedes Bermejo Herrero, Maria Esther Calonge Errejon, Laura Capa Muñoz, Maria Teresa Coiras Lopez, Francisco Diez Fuertes, Javier Garcia Perez, Nuria Gonzalez Fernandez, Elena Mateos De Las Moreras, Maria Teresa Perez Olmeda, Victor Sanchez Merino, Maria Eloisa Yuste Herranz.
